# Coronary Artery Calcium Is Independently Associated with Arterial Stiffness and LDL Cholesterol Burden in Patients with Familial Hypercholesterolemia

**DOI:** 10.3390/jcm14041245

**Published:** 2025-02-13

**Authors:** Alessandro Mattina, Antonina Giammanco, Davide Noto, Giulio Geraci, Emilio Nardi, Carlo Maria Barbagallo, Carola Maria Gagliardo, Maria Ausilia Giusti, Francesco D’Ignoto, Francesco Giallauria, Carla Di Benedetto, Antonella Maria Cardella, Patrizia Toia, Ludovico La Grutta, Angelo Baldassare Cefalù, Maurizio Averna

**Affiliations:** 1 Diabetes Service, Istituto Mediterraneo Per i Trapianti e Terapie ad Alta Specializzazione (IRCCS ISMETT), UPMC Italy, 90127 Palermo, Italy; alessandromattina@gmail.com (A.M.);; 2 Department of Health Promotion, Mother and Child Care, Internal Medicine and Medical Specialties (ProMISE), Azienda Ospedaliera Universitaria Policlinico Paolo Giaccone, University of Palermo, 90127 Palermo, Italy; 3 Department of Medicine and Surgery, “Kore” University of Enna, 94100 Enna, Italy; 4 Nephrology Unit, Istituto Mediterraneo Per i Trapianti e Terapie ad Alta Specializzazione (IRCCS ISMETT), UPMC, 90127 Palermo, Italy; 5 Department of Translational Medical Sciences, Federico II University of Naples, 80131 Naples, Italy; 6 UOS Terapia Semintensiva, PO Garibaldi Centro, ARNAS Garibaldi, 95123 Catania, Italy; 7 Department of Radiology, Azienda Ospedaliera Universitaria Policlinico Paolo Giaccone, 90127 Palermo, Italy; 8 Institute of Biophysics (IBF), National Research Council CNR, 90146 Palermo, Italy

**Keywords:** pulse wave velocity, familial hypercholesterolemia, coronary artery calcium, dyslipidemia, cardiovascular disease, vascular inflammation, cardiovascular risk, statin treatment, high-density lipoprotein, low-density lipoprotein

## Abstract

**Background:** Familial hypercholesterolemia (FH) is a genetic disorder characterized by high plasma levels of low-density lipoprotein cholesterol (LDL-C) and exposing patients to higher risk of early cardiovascular (CV) atherosclerotic diseases. Though the estimated prevalence of heterozygous FH (HeFH) is about 1 in 200, FH is still underdiagnosed and undertreated. Coronary artery calcification (CAC) assessment and arterial stiffness measured as pulse wave velocity (PWV) have demonstrated their accuracy in CV risk assessment, but data on HeFH are lacking. This study aims to evaluate CAC and PWV in a population of HeFH patients to improve risk stratification and therapy timing and setting. **Methods:** One hundred genetically characterized HeFH patients, regularly followed up since diagnosis, were recruited at our outpatient clinic. In all patients, CAC, PWV measurement, and LDL-C burden calculation were assessed. **Results**: The mean age was 45 ± 16 years. A total of 25% of patients had hypertension, and 15% were in secondary prevention. Through univariate analysis, we found strong positive correlations between CAC and both PWV (r = 0.52 *p* > 0.0001) and total LDL-C burden (r = 0.52 *p* < 0.0001). No other associations with lipid parameters were found. Multivariate analysis showed that CAC was independently associated with PWV adjusted for sex, total LDL-C burden, systolic blood pressure, smoking, LDL-C, HDL-C, and statin treatment. **Conclusions**: Arterial stiffness is strongly associated with CAC in HeFH patients with similar total LDL-C burden and CV risk profiles. Personalized risk assessment based on arterial stiffness and CAC evaluation enhances the stratification and management of cardiovascular risk in FH patients, supporting individualized therapeutic approaches.

## 1. Introduction

Dyslipidemia represents one of the most critical conditions associated with non-communicable diseases (NCDs), serving as a major risk factor for cardiovascular diseases (CVDs) [1]. NCDs, including cardiovascular diseases, diabetes, chronic respiratory diseases, and cancer, are responsible for more than 70% of global deaths, with dyslipidemia playing a central role in their pathogenesis. Elevated levels of low-density lipoprotein cholesterol (LDL-C) contribute significantly to atherosclerosis, leading to progressive vascular dysfunction and increased cardiovascular morbidity and mortality [2].

Familial hypercholesterolemia (FH, OMIM #143890) is an autosomal dominant genetic disorder caused by mutations in the genes coding for the low-density lipoprotein receptor (LDLR), apolipoprotein B (APOB), and pro-protein convertase subtilisin/kexin 9 (PCSK9) [3]. Individuals with heterozygous familial hypercholesterolemia (HeFH) are exposed to lifelong high LDL-C levels and are particularly vulnerable to cardiovascular (CV) events due to the development of atherosclerotic plaques [4]. The prevalence of HeFH is likely much higher than previously estimated, with recent studies suggesting it may affect as many as 1 in 200 individuals in the general population [5]. Despite its relatively high prevalence, HeFH remains underdiagnosed and undertreated, highlighting the need for improved screening strategies. If untreated, these patients have a dramatic increase in the risk of developing premature coronary heart disease [6], as well as peripheral artery disease [7] and carotid atherosclerosis [8].

The structural modifications in the arterial wall that occur in patients affected with atherosclerosis cause a profound alteration of the elastic properties of the vessels [9]. This process leads to a reduction in arterial compliance and increased stiffness [10]. Arterial stiffness is a well-established marker of vascular aging and is strongly associated with adverse cardiovascular outcomes. The pathophysiological mechanisms underlying arterial stiffening involve endothelial dysfunction, chronic inflammation, oxidative stress, and extracellular matrix remodeling, all of which are exacerbated in HeFH patients due to persistent hypercholesterolemia [11]. Arterial pulse wave velocity (PWV) measurement is an indirect method to evaluate arterial stiffness [12]. Carotid–femoral pulse wave velocity (cf-PWV) has been widely used to detect early stages of arteriosclerosis. Cf-PWV has been established as a reliable predictor of CVDs and may also serve as a valuable prognostic tool in the context of acute coronary syndrome [13]. Indeed, recent data coming from a meta-analysis of PWV including six studies have demonstrated the efficiency of this technique in predicting cardiovascular and all-cause mortality, especially when carotid–femoral PWV is considered [14].

The development of coronary artery calcification (CAC) is an active cellular process similar to bone mineralization and is an integral part of the atherosclerotic process. The calculation of CAC detected by a cardiac computed tomography (CT) scan without contrast medium has been found to be a safe and non-invasive technique. The Agatston score is the most used method that has been shown to correlate with an increased risk of cardiovascular diseases [15,16]. In the general population, both a high CAC score and a high PWV are associated with increased cardiovascular risk and the combination of a high CAC score and high PWV has been shown to be more predictive of cardiovascular events than either measure alone. This suggests that both the presence of plaque and the stiffness of the arteries contribute independently to the risk of future cardiovascular diseases [17].

As an added value, to achieve a more accurate estimation of lifelong exposure to elevated plasma cholesterol levels, an approach has been proposed to assess the cumulative LDL cholesterol burden (LDL-CB). LDL-CB has been linked to arterial stiffness [7] as well as coronary and aortic calcifications in the HeFH population [18,19].

HeFH serves as a paradigmatic example of how personalized medicine is crucial for cardiovascular risk stratification and for tailoring therapeutic strategies accordingly. The objective of this study was to evaluate cardiovascular risk in HeFH patients using personalized biomarkers, such as arterial stiffness, assessed via cf-PWV, and coronary artery calcification (CAC), detected using a CT scan and quantified using the Agatston score. These tools aim at supporting individualized therapeutic decisions and improving clinical management.

## 2. Materials and Methods

### 2.1. Study Population

One hundred patients were consecutively recruited at the Outpatient Lipid Clinic (Department of Health Promotion Sciences, Maternal and Infant Care, Internal Medicine and Medical Specialties) between February 2016 and November 2017. Only patients with a genetic diagnosis of HeFH at regular follow-up since the time of diagnosis were eligible for enrolment in this study.

The exclusion criteria included refusal to provide informed consent, contra-indication to CT scan, and uncontrolled hypertension or triglycerides (TG) > 400 mg/dL (4.52 mmol/L). All patients underwent routine clinical examinations and laboratory tests. Carotid–femoral PWV was assessed using SphygmoCor^®^ System Devices (AtCor Medical Pty Ltd. Sydney, Australia), XCEL Software Suite Version 1.1, outlined in the relative section.

The study was carried out according to the principles outlined in the Declaration of Helsinki. Approval from the local Ethics Committee was obtained, and informed consent was signed by all participants.

### 2.2. Clinical Parameters, Traditional CV Risk Factors, and Therapy Management

History of ischemic heart disease, stroke, severe peripheral vasculopathy (indicated as secondary prevention), arterial hypertension (AH), and type 2 diabetes (T2D) were assessed using the patients’ clinical records. Cigarette smoking (yes/no), body mass index (BMI) (weight in kilograms divided by the square of height in meters), and presence of xanthomas (yes/no) were also evaluated. Therapy escalation to ezetimibe or PCSK9 inhibitors followed the ESC/EAS guidelines for the management of dyslipidemias, with LDL-C goals defined according to cardiovascular risk levels [20]. The escalation approach aimed to achieve ≥50% LDL-C reduction from baseline and meet LDL-C goals (<55 mg/dL for very high CV risk, <70 mg/dL for high CV risk, and <100 mg/dL for moderate CV risk) while respecting patient tolerance.

### 2.3. CT Scan Parameters

The calcium score of coronary arteries was evaluated by cardiac CT scan without contrast medium. The CAC was performed using a 128-layer scanner (Definition AS+, Siemens Healthcare, Forchheim, Germany) with standard protocol (slice thickness 3 mm, perspective gating, and caudal skull extension from the tracheal hull to the diaphragm). The image datasets were transferred to a dedicated workstation (Leonardo, Siemens, Germany) with specific post-processing software (Syngo Via Cardiac version VA20A, Siemens, Germany). The images were evaluated by a single radiologist with extensive expertise in cardiac CT. Coronary calcium was quantified using the Agatston method. The radiation dose given to patients was as low as <1.5 mSv.

### 2.4. Carotid Intima-Media Thickness Assessment

Carotid ultrasound examination was performed by a single experienced operator following the recommendations of the Mannheim Carotid Intima-Media Thickness Consensus [21]. Six measurements were obtained on each side, and both the mean and maximum carotid intima-media thickness (cIMT) values were considered. In the presence of carotid plaques, cIMT was measured proximally at a plaque-free site. A mean cIMT value > 0.90 mm was considered abnormal, in accordance with the ESC/ESH guidelines for hypertension management [22].

### 2.5. Arterial Stiffness Evaluation

The pulse wave velocity was assessed using a SphygmoCor^®^ System Device and a single investigator. After measuring brachial blood pressure, sequential applanation tonometry of the carotid and femoral arteries was performed, with simultaneous electrocardiogram (ECG) signal recording. The difference between carotid and femoral blood transit times divided by the estimation of the arterial path length allowed for the calculation of the cf-PWV. The SphygmoCor^®^ cf-PWV is a validated non-invasive hemodynamic measurement device recommended for the indirect evaluation of arterial stiffness [23].

### 2.6. Lipid Analysis and Genetic Assessment

Blood samples from patients (10 mL each) were collected in both plain and EDTA-containing (1 mg/mL) tubes. Plasma TC, TG, and HDL-C were measured using standard enzymatic–colorimetric procedures (Instrumentation Laboratory, NY, USA) in ILAB 300 Plus auto-analyzer Clinical Chemistry System (Instrumentation Laboratory, NY, USA). LDL-C was calculated using the Friedewald formula only for patients with TG levels < 250 mg/dL [24]. The candidate genes for FH were analyzed as previously described [25,26].

### 2.7. Cholesterol Burden Calculation

The cumulative LDL cholesterol load (cholesterol burden) was estimated through the calculation of total LDL cholesterol burden (mg-years/dL) according to Hoeg et al. [27]. Total LDL cholesterol burden (LDL-CB) is the sum of LDL cholesterol burden at diagnosis (dLDL-CB) and cholesterol burden post diagnosis (pdLDL-CB). The dLDL-CB is obtained by multiplying the initial serum LDL cholesterol (LDL-C) value by the patient’s age at the time of diagnosis. The pdLDL-CB is calculated by summing the LDL-C values dosed annually during the follow-up, using patient files.

### 2.8. Statistical Analysis

All continuous variables are reported as mean ± standard deviation (SD) for parametric variables and median (interquartile range) for nonparametric variables, and as frequency (percentage) for categorical variables. Correlations between two variables were assessed using a linear regression model, utilizing either Pearson’s correlation coefficient (r) or Spearman’s rho for nonparametric variables. The differences between groups were evaluated using the Student’s *t*-test for continuous variables, and χ^2^ test for categorical variables. Multivariate linear regression analysis was used to assess the independent contribution of the variables. Age was not considered separately, as it was already included in the LDL-CB equation. A *p* value < 0.05 was considered significant. JMP^®^ Statistical Software, Version 11, SAS Institute Inc., Cary, NC was used for statistical calculations.

## 3. Results

In this study, we enrolled one hundred genetically diagnosed HeFH patients (50 males and 50 females). Table 1 shows the cohort’s clinical data. The mean age of the studied HeFH population was 45.9 ± 16.2 years, and the mean age of lipid lowering treatment (LLT) was 29.3 ± 14.2 years.

A total of 25% percent of these patients also had hypertension, and 24% were on treatment for this condition. Seven patients (7%) had T2D, and 31% were smokers. Only 13% of these patients were in secondary prevention. Supplementary Table S1 summarizes the cardiovascular risk stratification and the proportion of patients achieving their LDL-C goals according to their risk category, based on the ESC/EAS guidelines [20]. Specifically, 41% of patients were classified as low risk, 19% as moderate risk, 9% as high risk, and 31% as very high risk. Overall, 43% of patients achieved their LDL-C target during the study period. The assessment of carotid atherosclerosis revealed a generally higher cIMT than expected for the patients’ age. Specifically, the mean maximum cIMT was 0.8 mm.

Additionally, 88% of patients were receiving statin therapy, 45% were treated with ezetimibe, and 8% were on the PCSK9 monoclonal antibody alirocumab. The statins prescribed included rosuvastatin, atorvastatin, simvastatin, pravastatin, and lovastatin. Detailed information about the number of patients undergoing treatment with each statin and the corresponding dosage is provided in Supplementary Table S2. The patients who did not receive statin therapy reported reasons such as intolerance, refusal to adhere to therapy, or lack of access to PCSK9 inhibitors at the time of the study.

**Figure 1:**
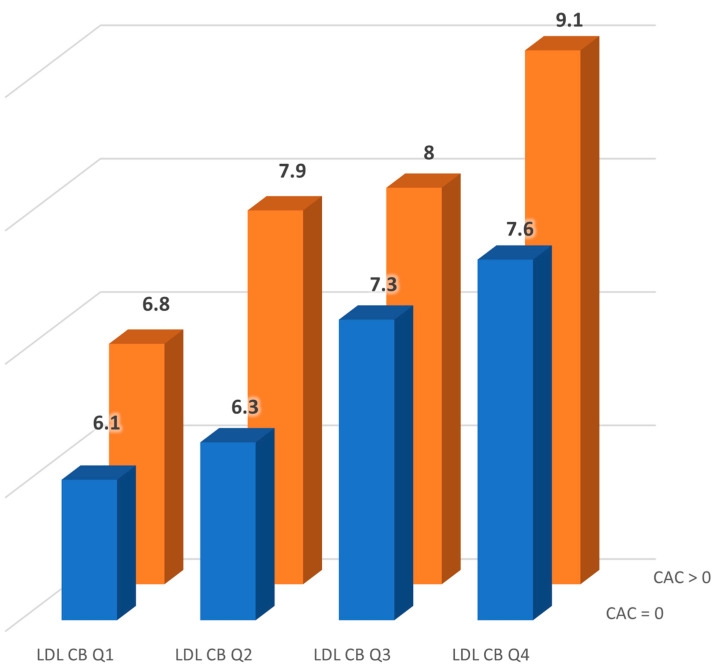
Pulse wave velocity (m/s) across low-density lipoprotein cholesterol burden (LDL-CB) groups stratified by the presence or absence of coronary artery calcification (CAC). LDL-CB groups were defined by quartiles of total LDL-CB (Q1–Q4).

Table 2 and Figure 1 show the correlation of CAC with the study parameters. CAC was positively correlated with PWV (r = 0.52 *p* < 0.0001) and total LDL-C burden (r = 0.52 *p* < 0.0001) on univariate analyses. CAC was also correlated with classical CV risk factors such as age (r = 0.65 *p* < 0.0001), type 2 diabetes (r = 0.38 *p* < 0.0001), and hypertension (r = 0.51 *p* < 0.0001). No associations with other lipid parameters were found. Furthermore, we found that male sex, current smoking, and statin therapy did not correlate with CAC in these patients. A significant linear correlation was observed between both maximum (r = 0.43, *p* < 0.0001) and mean (r = 0.48, *p* < 0.0001) cIMT and CAC.

Multivariate analysis (Table 3) showed that CAC was independently associated with PWV adjusted for sex, total LDL-C burden, hypertension, smoking, LDL-C, and HDL-C.

## 4. Discussion

The present study showed that arterial stiffness was independently associated with CAC in HeFH patients who exhibited a similar LDL-C burden and CV risk profile. These findings suggest a strong and clear correlation between CAC and PWV, as well as between CAC and cumulative LDL-C burden.

Patients with FH have high lifelong exposure to LDL cholesterol levels, and the process of arterial stiffening could therefore be more extensive, not only affecting the muscular, but also the elastic arteries [28]. Hypercholesterolemia due to LDL receptor impairment in FH patients has a potential role in directly determining arterial inflammation and calcium deposition in the vascular wall of young subjects [29]. This may explain the increased arterial stiffness.

Cf-PWV has been shown to correlate with subclinical target organ damage, reinforcing its role in refining cardiovascular risk stratification beyond traditional risk factors. Previous studies have reported stiffening of the arteries in patients at increased risk for atherosclerotic diseases, including hypercholesterolemic patients [30,31,32,33,34]. A meta-analysis of 8 studies, involving 317 FH patients matched with 244 normocholesterolemic individuals, reported no significant differences in PWV values between the 2 groups [35]. Despite this, PWV is widely recognized as a strong predictor of cardiovascular and all-cause mortality. Numerous studies investigating PWV within the FH population have highlighted significant associations with various vascular parameters [14]. Associations between atherosclerosis and arterial stiffness in FH patients has also been evaluated in case–control studies. Cheng et al. [18] found a correlation between cholesterol burden, carotid intima-media thickness (cIMT), and PWV in a small group of FH patients. Furthermore, the augmentation index (AIx) has been proposed as an independent determinant of cIMT and a potential vascular risk marker for FH management [36].

Martinez et al. [37] evaluated several atherosclerotic and lipid parameters, including CAC, LDL year score (LYS), and cIMT. The authors questioned the relevance of imaging and plasma inflammatory markers in predicting coronary heart disease (CHD) in FH patients. Nevertheless, they calculated the LYS simply by multiplying the age in years by the LDL-C level, a method that does not account for pre-treatment LDL-C levels or their lifelong high variability. Despite these methodological challenges, independent correlations have been observed between cIMT and PWV, supporting the relationship between arterial stiffness and cardiovascular risk in this population. Tada et al. suggested that arterial stiffness assessed using PWV is significantly associated with CHD in patients with FH [38]. In adults with FH, no relationship between arterial compliance and classical lipid variables was found [39]. This suggests that non-lipid factors, such as genetic predisposition to vascular remodeling, low-grade inflammation, and altered nitric oxide bioavailability may play an equally important role in arterial stiffness progression. The evidence also underscores the critical role of cholesterol burden in the pathophysiology of arterial structure and function [18]. Notably, carotid and femoral artery wall stiffness and thickness have shown significant improvement after one year of lipid-lowering therapy in FH patients [32]. These findings underscore the importance of early and aggressive lipid-lowering strategies, as delaying treatment may allow for irreversible vascular changes to progress.

Coronary calcium imaging detects the calcium burden, and cannot identify a vulnerable plaque, but it may be able to globally define the risk of a patient’s coronary events [40,41]. An increased arterial wall stiffness might enhance the risk of plaque rupture and may eventually contribute to acute ischemic events [42].

To the best of our knowledge, this is the first study to evaluate the relationship between CAC, pulse wave velocity, and LDL cholesterol burden in primary and secondary prevention FH patients receiving conventional lipid-lowering therapy. Through multivariate analysis, we found that in FH patients with similar LDL-C burden and similar traditional CV risk factors, coronary artery calcium is strongly associated with increased arterial stiffness. This confirms that other factors exist besides the lipid parameters involved in atherosclerotic damage in FH patients, linked to the elastic properties and inflammation of the vessels. The cardiovascular risk in these patients is difficult to define. Markers of arterial stiffness and coronary atherosclerosis can be regarded as valuable tools for personalized CV risk stratification in this specific patient population. This approach can improve disease management and aligns with personalized prevention strategies for CVDs, which are increasingly demanding in addressing chronic conditions and other NCDs.

This study presents some limitations: PWV reproducibility is rather low and is age- and blood-pressure-dependent. However, it is a non-invasive, validated tool for easily discriminating vascular impairment, especially in a homogeneous and mostly young sample of patients like ours. Furthermore, PWV assessment was performed by a single clinician avoiding inter-operator variability. Although the sample size was relatively small, the calculation of LDL cholesterol burden required an accurate selection of patients followed since FH diagnosis. It would have been useful to perform separate analyses for the primary and secondary prevention groups; however, our population, predominantly composed of relatively young patients, had a limited number of patients in secondary prevention. As a result, separate analyses did not yield statistically significant findings for this subgroup. Nevertheless, we conducted analyses for the primary study outcomes, corrected for the type of prevention (Supplementary Table S3), and a multivariate model including secondary prevention (Supplementary Table S4), and the results remained consistent with the overall cohort. In addition, there was no control group that could strengthen the study outcomes. However, the LDL cholesterol burden was a pivotal variable for the study purposes, and its calculation was unreliable in non-FH control patients.

## 5. Conclusions

This study confirms the role of cardiovascular risk determinants of CAC in primary and secondary prevention FH patients. Our data also suggest that arterial stiffness could reflect the presence and severity of coronary impairment in patients with FH by identifying those who have a higher atherosclerotic burden despite having the same LDL cholesterol burden. Markers of arterial stiffness and coronary atherosclerosis enhance personalized cardiovascular risk stratification of FH patients and can consequently be considered a support option for adopting appropriate therapeutic choices.

## Figures and Tables

**Table 1:** Clinical parameters of the FH cohort.

Clinical Characteristics		Total Population
	n	100
	Age, years	45.9 ± 16.2
	Sex, male (%)	50 (50)
	PWV, m/s	7.44 ± 2.36
	Xanthomas, n (%)	21 (21)
	Age at start of treatment, years	29.3 ± 14.2
	Systolic BP, mmHg	128.8 ± 21.4
	Diastolic BP, mmHg	73.2 ± 11.3
	Mean BP, mmHg	93.7 ± 13.0
**Cardiovascular risk factors**		
	Secondary prevention, n (%)	13 (13)
	Current smoking, n (%)	31 (31)
	Type 2 Diabetes, n (%)	7 (7)
	BMI, kg^2^/h	27.4 ± 4.9
	Arterial hypertension, n (%)	23 (23)
**Treatment**		
	Antihypertensive treatment, n (%)	24 (24)
	Statins, n (%)	88 (88)
	Ezetimibe, n (%)	45 (45)
	Alirocumab, n (%)	8 (8)
**Lipid profile**		
	Total cholesterol, mmol/L	5.7 (4.8–7.1)
	LDL cholesterol, mmol/L	3.7 (2.8–4.7)
	HDL cholesterol, mmol/L	1.4 (1.2–1.6)
	Triglycerides, mmol/L	1.0 (0.7–1.6)
	Non-HDL cholesterol, mmol/L	4.2 (3.3–5.4)
	Lp(a), mg/dL	18.8 (5.52–47)
**LDL-C burden**		
	LDL cholesterol burden pre-treatment, mmol-years/L	181.4 (133.7–286.6)
	LDL cholesterol burden post treatment, mmol-years/L	70.9 (33.7–111.5)
	LDL cholesterol burden total, mmol-years/L	259.7 (186.6–338.7)
**Carotid atherosclerosis**		
	Maximum cIMT, mm	0.8 (0.63–0.90)
	Mean cIMT, mm	0.7 (0.60–0.85)
**CAC**		
	Agatston calcium score, log	2.14 ± 2.69

PWV: pulse wave velocity; BMI: body mass index; BP: blood pressure; LDL: low-density lipoprotein; HDL: high-density lipoprotein; Lp(a): lipoprotein(a); cIMT: carotid intima-media thickness; and CAC: coronary artery calcium.

**Table 2:** Correlations between coronary artery calcium log (logCAC) and main parameters in whole population.

		logCAC	
		r	*p*
**Characteristics**			
	Age, years	0.65	<0.0001
	Sex, male	0.14	0.1803
	PWV, m/s	0.52	<0.0001
	Xanthomas	0.26	0.0104
**Cardiovascular risk factors**			
	Secondary prevention	0.45	<0.0001
	Current smoking	−0.05	0.6560
	Type 2 Diabetes	0.38	<0.0001
	BMI, kg^2^/h	0.28	0.0044
	Arterial Hypertension	0.51	<0.0001
	Systolic BP, mmHg	0.46	<0.0001
	Diastolic BP, mmHg	0.21	0.0381
	Mean BP, mmHg	0.37	0.0001
**Treatment**			
	Antihypertensive treatment	0.49	<0.0001
	Statins	−0.09	0.3509
	Ezetimibe	0.28	0.0051
	Alirocumab	0.27	0.0059
**Lipid profile**			
	Total cholesterol, mmol/L	−0.10	0.3051
	LDL cholesterol, mmol/L	−0.12	0.2420
	HDL cholesterol, mmol/L	−0.07	0.4968
	Triglycerides, mmol/L	0.12	0.2536
	Non-HDL cholesterol, mmol/L	−0.09	0.3564
	Lp(a), mg/dL	−0.09	0.6342
**LDL-C burden**			
	LDL cholesterol burden pre-treatment, mmol-years/L	0.49	<0.0001
	LDL cholesterol burden post treatment, mmol-years/L	0.23	0.0228
	LDL cholesterol burden total, mmol-years/L	0.55	<0.0001
**Carotid atherosclerosis**			
	Maximum cIMT, mm	0.43	<0.0001
	Mean cIMT, mm	0.48	<0.0001

PWV: pulse wave velocity; BMI: body mass index; BP: blood pressure; LDL: low-density lipoprotein; HDL: high-density lipoprotein; and Lp(a): lipoprotein(a).

**Table 3:** Variables independently associated with coronary artery calcium Log (logCAC).

	Log CAC		
	R^2^	β (SE)	*p*
**Overall Model**	0.48		<0.0001
**PWV (m/s)**		0.337 (0.109)	0.0026
**Total LDL-C Burden (mmol/L)**		0.009 (0.002)	<0.0001
**Sex (male)**		0.820 (0.486)	0.0954
**Mean Blood Pressure (mmHg)**		0.013 (0.019)	0.5004
**Smoking (yes = 1)**		0.200 (0.454)	0.6597
**LDL-C (mmol/L)**		−0.007 (0.004	0.0993
**HDL-C (mmol/L)**		−0.018 (0.018)	0.3205
**Lipid-lowering drugs (yes = 1)**		−1.057 (0.750)	0.1622

PWV: pulse wave velocity; LDL-C: low-density lipoprotein cholesterol; HDL-C: high density lipoprotein cholesterol. Lipid-lowering drugs refer to statins and/or ezetimibe and/or alirocumab.

## Data Availability

The original contributions presented in the study are included in the article, further inquiries can be directed to the corresponding authors.

## Supplementary Materials

The following supporting information can be downloaded at: https://www.mdpi.com/article/10.3390/jcm14041245/s1.
