# Supplementary material for: Coronary Artery Calcium Is Independently Associated with Arterial Stiffness and LDL Cholesterol Burden in Patients with Familial Hypercholesterolemia
Source: J Clin Med. 2025 Feb 13;14(4):1245. doi: 10.3390/jcm14041245 (PMC11856069; doi:10.3390/jcm14041245)
Supplement: Supplementary file 1 [file jcm-14-01245-s001.zip › jcm-3452940-supplementary.pdf]

Supplementary Materials

Table S1. Distribution of cardiovascular risk and low-density lipoprotein cholesterol (LDL-C) goal achievement in whole population.

Risk Category	Patients (n)	Patients achieving LDL-C target (n)
**All**	100	43
**Low Risk**	41	21
**Moderate Risk**	19	7
**High Risk**	9	4
**Very High Risk**	31	11

Table S2. Patient distribution based on statin therapy and dosage regimen.

Statin	Dosage (mg)	Patients (n)
**Rosuvastatin**	**Overall**	**37**
	10	5
	20	27
	40	5
**Atorvastatin**	**Overall**	**27**
	10	5
	20	12
	40	9
	80	1
**Simvastatin**	**Overall**	**18**
	10	1
	20	7
	40	9
	60	1
**Pravastatin**	**40**	**5**
**Lovastatin**	**20**	**1**

Table S3. Correlations between coronary artery calcium (logCAC), adjusted for secondary prevention status, and outcome parameters in the whole population.

	logCAC	
	**β (SE)**	**p**
**PWV (m/s)**	0.502 (0.094)	< 0.0001
**Total LDL-C Burden (mmol/L)**	0.009 (0.002)	< 0.0001

PWV: pulse wave velocity and LDL-C: low density lipoprotein cholesterol.

Table S4. Variables independently associated with Coronary Calcium Score (logCAC).

	logCAC		
**Analysis**	**R^2**	**β (SE)**	**p**
**Overall Model**	0.53		< 0.0001
**PWV (m/s)**		0.298 (0.105)	0.0057
**Sex (men = 1)**		0.524 (0.473)	0.2714
**Total LDL-C Burden (mmol/L)**		0.008 (0.002)	< 0.0001
**Mean Blood Pressure (mmHg)**		0.011 (0.018)	0.5500
**Smoking (yes = 1)**		0.311 (0.434)	0.4763
**LDL-C (mmol/l)**		-0.005 (0.004)	0.1986
**HDL-C (mmol/l)**		-0.016 (0.018)	0.3592
**Lipid-lowering drugs (yes = 1)**		-0.834 (0.719)	0.2490
**Secondary prevention (yes = 1)**		1.978 (0.627)	0.0022

PWV: pulse wave velocity; LDL-C: low-density lipoprotein cholesterol; HDL-C: high-density lipoprotein cholesterol. Lipid-lowering drugs refer to statins and/or ezetimibe and/or alirocumab.
